# The Cry of the Children

**Published:** 1898-07-23

**Authors:** 


### MODERN SOCIOLOGY.

### "THE CRY OF THE CHILDREN."

Do we hear it again? Has not all been done that mortal power can do to prevent children having cause to cry out against the fate that sent them into this world? So some of us had thought; but Mr. Frank Hird's book* bearing the above title, contains facts that fully justify its name. It deals with industries in which children are employed in the East-end of London, in a fashion most inimical to both their development and their education. In box-making, belt and umbrella making, paper-bag and sack-making, artificial flower-making, and furniture-polishing, as well as on canal barges, he shows that children are employed when they ought to be at school; and, even when the fear of the School Board avails to send them to some place of education, it cannot prevent their being kept at work far into the night, when they ought to be at rest.

Mr. Hird's book has many merits. One is that it is founded on personal knowledge. Another is that it is a fair, and, so far as so pitiful a subject will permit, an unimpassioned statement of facts, without those accusations against society at large which prejudice one's judgment in the reading of books based on similar grievances. It is true that once, and once only, Mr. Hird declares that every woman who yields to the feminine impulse to buy a bargain encourages those sweated industries from which children suffer most. That is true; but it must not be forgotten that if womankind, with one consent, refrained from buying those cheap articles which sweated labour produces, it would not at once—though we believe it would ultimately—help the victims of underpaid work. Let us take the case of belt-making, in which Mr. Hird reckons the remuneration at three-farthings an hour. These belts may be sold at a shilling each. Suppose that the price of these was raised to one and threepence; and suppose also—which is a wild hypothesis—that the extra threepence was given to the worker, what would be the result? That fewer women would buy these belts. Some would do so—the comparatively few to whom the rise in price made little difference; others would recall that they had an old one at home that would "do"; others would get material, and try to make up the belt themselves; others would go without the belt altogether. The result would be, indeed, that beltmakers would be better paid, but fewer of them would be required. Some would get a whole loaf instead of a half one, but the rest would lack bread altogether. We need not go on to the ultimate result, that beltmaking being a better-paid industry than many others, so many workers would flock into it that the price of labour would fall again to the old level. The first result of any interference with wages is sufficient to show the fallacy of the *caveat emptor* which is so often hurled at our heads. We have none of us more than a certain amount of money to spend. If we have to pay more for a given article than we did before, we stop buying it, if it is an article of luxury; or if it is an article of necessity, we stop or limit our buying of something else. In either case some class of workers suffer. Herein lies the great difficulty of philanthropy, and the cause of the failure of all philanthropy which takes no account of economics.

Does this mean, then, that we shall sit down and take it as a divine ordinance that certain women and children—when you find the children at work that is ill-paid and onerous you may be sure that women are at it too—are for ever to lead lives destitute of all health, all joy, all fair reward of labour, in order that other people may have certain articles cheap? Surely not; but the remedy lies for the most part beyond the law, beyond the power of outsiders altogether. Mr. Hird owns that in almost all the cases he quotes, the children have to keep not only themselves, but drunken, lazy, loafing fathers. This is the crux of the difficulty. If these men worked they could probably keep themselves and their families; their children would be at school obtaining such an education as might fit them for better work than they now have any chance of ever learning; they would not be stunted and half-starved; they would have the chance of becoming good and useful citizens, which, as things go at present, seems rather beyond the bounds of possibility. But it is just herein that the trouble lies. The law does its best for the children, but it cannot control the parents. It can compel children to go to school, though it tasks all its wisdom to cope with the manifold evasions of mothers who need the child's aid to earn enough for the child's food; but it cannot prevent the time that is spent outside the school being employed in such work as sends the child back on the morrow too fatigued to profit by instruction. Yet one thing is possible. Work is not given out to children of school age. The nominal worker is the mother, or some young person, who works in a factory all day and takes home some work to finish at night. Very often this latter is more than she could by any possibility accomplish herself, and she "sub-contracts" it to children—her own or her neighbours'. If it were forbidden to employ a worker inside and outside a factory on the same day, this system would be almost completely ended. This, though he does not mention it, we may take as one moral to be drawn from Mr. Hird's book.

There is another, which is pointed by the remark of the little maker of artificial flowers who, when she was taken to the country, and saw, for the first time in her life, a bush with real roses upon it, wondered "if God was riled" with her and her fellow-workers, who made such travesties of His work. Mr. Hird notes here that the French flower-maker is trained to her work, educated to observe colouring, texture, shape, the result being that her products command a good price, and she herself is well paid; while the poor English worker supplies only the poorest market, where things are cheap, and the workers are paid in proportion. Does not this suggest that our educational system will not be complete until it furnishes more varied technical training, especially for girls, for whom, as a rule, only such knowledge as will fit them for housekeeping is provided. In France and Belgium there are regular trade schools, varying in different districts according to the prevailing industry, which afford technical training that can be promptly turned to practical account, which training largely accounts for the superiority of foreign products to our own in so many articles.

* "The Cry of the Children: An Exposure of Certain British Industries in which Children are Iniquitously Employed." By Frank Hird. Illustrated by D. Macpherson. London: James Rowden, 10, Henrietta Street, Covent Garden. Price 1s.

